# The environment and species affect gut bacteria composition in laboratory co-cultured *Anopheles gambiae* and *Aedes albopictus* mosquitoes

**DOI:** 10.1038/s41598-020-60075-6

**Published:** 2020-02-25

**Authors:** Sally A. Saab, Heinrich zu Dohna, Louise K. J. Nilsson, Piero Onorati, Johnny Nakhleh, Olle Terenius, Mike A. Osta

**Affiliations:** 1 Department of Biology, American University of Beirut, Bliss Street, Beirut, Lebanon; 2 Department of Ecology, Swedish University of Agricultural Sciences (SLU), Box 7044, SE-750 07 Uppsala, Sweden; 3 Department of Cell and Molecular Biology, Microbiology, Uppsala University, BMC, Box 596, SE-75124 Uppsala, Sweden

**Keywords:** Microbiome, Entomology

## Abstract

The midgut microbiota of disease vectors plays a critical role in the successful transmission of human pathogens. The environment influences the microbiota composition; however, the relative mosquito-species contribution has not been rigorously disentangled from the environmental contribution to the microbiota structure. Also, the extent to which the microbiota of the adult sugar food source and larval water can predict that of the adult midgut and *vice versa* is not fully understood. To address these relationships, larvae and adults of *Anopheles gambiae* and *Aedes albopictus* were either reared separately or in a co-rearing system, whereby aquatic and adult stages of both species shared the larval water and sugar food source, respectively. Despite being reared under identical conditions, clear intra- and interspecies differences in midgut microbiota-composition were observed across seven cohorts, collected at different time points over a period of eight months. Fitting a linear model separately for each OTU in the mosquito midgut showed that two OTUs significantly differed between the midguts of the two mosquito species. We also show an effect for the sugar food source and larval water on the adult midgut microbiota. Our findings suggest that the mosquito midgut microbiota is highly dynamic and controlled by multiple factors.

## Introduction

The mosquito gut microbiota has a significant impact on several physiological processes of the host including the regulation of basal immunity in the gut^1,2^, the synthesis of the peritrophic matrix^3^, development^4^, and transmission of human pathogens^1^. It has become increasingly clear that the midgut microbiota significantly influences the vectorial capacity of mosquitoes. In *Anopheles gambiae*, removal of the microbiota by antibiotic treatment rendered mosquitoes more susceptible to *Plasmodium* infections^1,5^. This phenotype is to a significant extent due to the role of the microbiota in increasing basal level expression of immune effector molecules active against both bacteria and *Plasmodium* parasites^1^. Some members of the microbiota have been shown to compromise the survival of *Plasmodium* parasites directly, independent of the host^6^. Studies in the major dengue vector *Aedes aegypti* also revealed a significant role of the microbiota in reducing vector competence for dengue virus^2,7^.

Previous studies on the mosquito gut-microbiota revealed significant differences in the microbiota composition between different species, but also between individuals of the same species, rendering it difficult to assign a core microbiota. They also showed that the midgut microbiota is usually dominated by few phyla^8–11^. In *Anopheles*, analysis of the microbiota in 25 wild-caught mosquitoes of *An. gambiae* and *An. coluzzii* from Cameroon revealed that the large majority of bacteria in adult midguts belonged to Proteobacteria while in larval midguts this phylum was less represented. In fact, the midguts of newly emerged *An. coluzzii* and *An. gambiae* adults were mainly colonized by four prominent classes: Gammaproteobacteria, Alphaproteobacteria, Betaproteobacteria and Actinobacteria^12^. Similarly, a study by Boissiere *et al*. revealed that the midguts of adult *An. gambiae* mosquitoes collected from their natural breeding sites in Cameroon were dominated by the same four classes with Actinobacteria being relatively less abundant^8^. Interestingly, there were drastic differences between the midgut microbiota of field-collected and lab-reared mosquitoes; the former was more diverse and constituted mainly of Proteobacteria while the latter showed less diversity and was dominated in particular by the Flavobacteria *Elizabethkingia spp*.^8^.

In *Aedes*, on the other hand, the analysis of total microbiota from adult field-caught and lab-reared *Ae*. *albopictus* mosquitoes showed that both were dominated by Proteobacteria and share other less abundant phyla^13^. Characterization of the midgut microbiota of *Ae. aegypti* mosquitoes using culture-dependent and culture-independent techniques revealed also the presence of a core microbiota, whereby *Pseudomonas* was the most abundant genus constituting around 70% of midgut bacteria in wild-caught females^14^. Interestingly, the microbiota structure was similar between lab-reared and field-caught mosquitoes suggesting that the *Ae. aegypti* gut might constitute a competitive environment^14^. In fact, when lab-reared *Ae. aegypti* mosquitoes were fed gut bacteria isolated from humans, frogs, *An. gambiae*, and *Ae. aegypti*, the long-time persistence of these bacteria in the mosquitoes was dependent on host origin. Even the same species of bacteria (*Pantoea stewartii*) survived better in *Ae. aegypti* when isolated from *Ae. aegypti* than when isolated from *An. gambiae* suggesting that specific host-bacteria co-adaptation is a prerequisite for bacteria to persist as components of the microbiota^15^.

**Figure 1:**
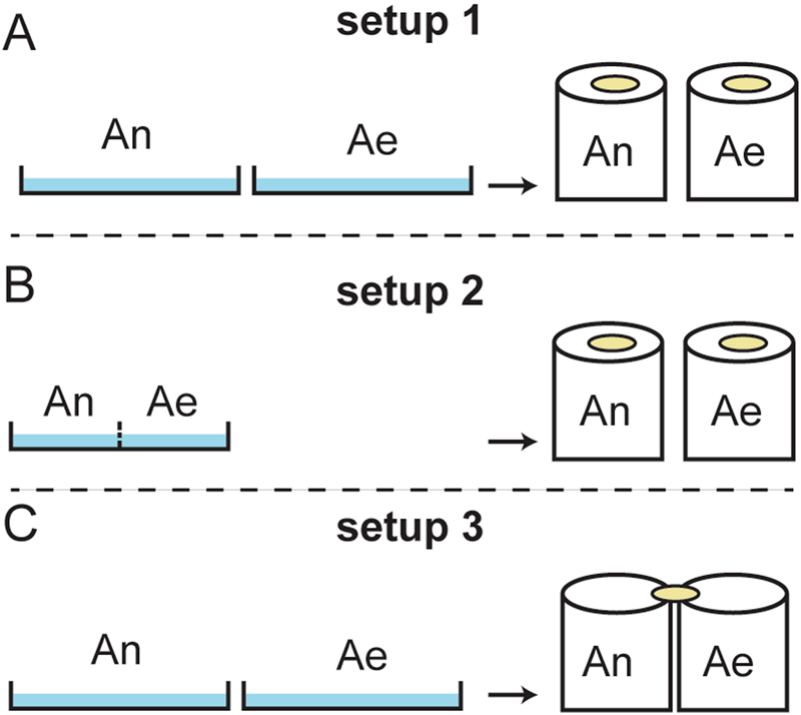
The different mosquito culturing setups. (**A**) The two mosquito species reared independently in separate larval pans and in separate cages. (**B**) The two species co-reared in the same pan (but separated by a net to avoid predation) to analyze the effect of sharing the same larval water on the adult midgut microbiota. (**C**) The two species reared independently from each other, but adults of both species share the same sugar source in order to analyze the effect of sharing the sugar source on the adult midgut microbiota. An=*Anopheles gambiae*, Ae=*Aedes albopictus*.

The fact that field-caught mosquitoes generally show more diversity in their midgut microbiota-composition highlights the important role of the environment in shaping the microbiota^8,11^. The strength of the environment in shaping the midgut microbiota is also reflected in a recent study in which geographically diverse colonies of *Ae. aegypti* harbored a similar midgut microbiota when reared in the same insectary^16^. Bacterial content of adult mosquito midguts may be acquired by feeding on nectar^17^, imbibing water from larval habitats during emergence^18^, or transstadially from larval gut bacteria during metamorphosis^4,19^. Hence, the breeding site from where mosquitoes are collected is likely to influence the adult midgut microbiota. However, there is conflicting data in the literature regarding this point. In certain studies, the sampling locality or breeding site showed no significant correlation with the midgut microbiota composition^10,11^, while in others a positive correlation was detected^8,12,20^. While most of the existing studies relied mainly on measuring the impact of the larval habitat microbiota on the composition of that of the adult midgut, the contribution of the adult vegetative food-source microbiota remains unclear. Male and female adult mosquitoes feed on natural sugar sources such as flower nectar, honeydew and fruits for energy and nutrition^21,22^. The natural sugar source seems to have an important impact on mosquito population size, survival rates and gonotrophic cycles^23,24^. Here, we wanted to address the extent to which the microbiota of the adult food source (i.e. sugar source) and larval water can predict that of the adult midgut and *vice versa* in a co-culture system of *An. gambiae* and *Ae. albopictus* that also allows for measuring the species effect on midgut microbiota-composition. In this system, the aquatic stages of both species were either reared separately or co-cultured in the same pans. Co-cultured larvae were physically separated by a porous mesh to avoid predation by one species on the other, but allowing the larval water to flow freely. Adult mosquitoes of both species, while being physically separated in independent cages, were either allowed to share the same sugar pad or feed on independent pads (Fig. 1). Our data revealed that despite being reared in the same insectary and given the same food source, significant intraspecies and interspecies variations in the midgut microbiota composition were observed across cohorts. We also show that both larval water and adult sugar food source contribute to midgut microbiota composition in adult mosquitoes.

## Results

### Overall bacterial community composition

MiSeq sequencing of the 16S rDNA amplicons generated 5,172,291 sequences after merging the paired end reads. Reads per pool of samples ranged between 681,657 and 1,160,434. After quality filtering and removal of non-16S rDNA sequences, the number of retained reads was 2,442,545; out of these, 230,219 reads corresponded to 27 larval water samples, 293,568 to the 33 sugar samples, 878,838 to the 96 *An. gambiae* midguts and 1,039,920 to the 100 *Ae. albopictus* midguts. The total number of operational taxonomic units (OTUs) identified at the 97% cut-off threshold was 967. Sample complexity for each type of sample taken across all the seven different cohorts was as follows: 177 OTUs identified in larval waters; 106 in sugar pads; 708 in *An. gambiae* midguts and 791 in *Ae. albopictus* midguts.

The 967 OTUs were assigned to 25 bacterial phyla, except 41 OTUs which could not be assigned to any phylum (Supplementary Table S1). Of those assigned, 81% belonged to five phyla: Proteobacteria (32.8%), Firmicutes (16.6%), Bacteroidetes (13.2%), Actinobacteria (12.4%) and Parcubacteria (6%). The 3 most abundant phyla in the larval water were: Bacteroidetes (41%), Proteobacteria (35%) and Actinobacteria (21%). However, the phylum Proteobacteria was dominant in sugar pads (99%), *Ae. albopictus* midguts (90%) and *An. gambiae* midguts (63%) (Supplementary Table S1). Bacteroidetes was the second most abundant phylum (30%) in *An. gambiae*.

### The effect of cohort, food source and species on the midgut microbiota-composition

**Figure 2:**
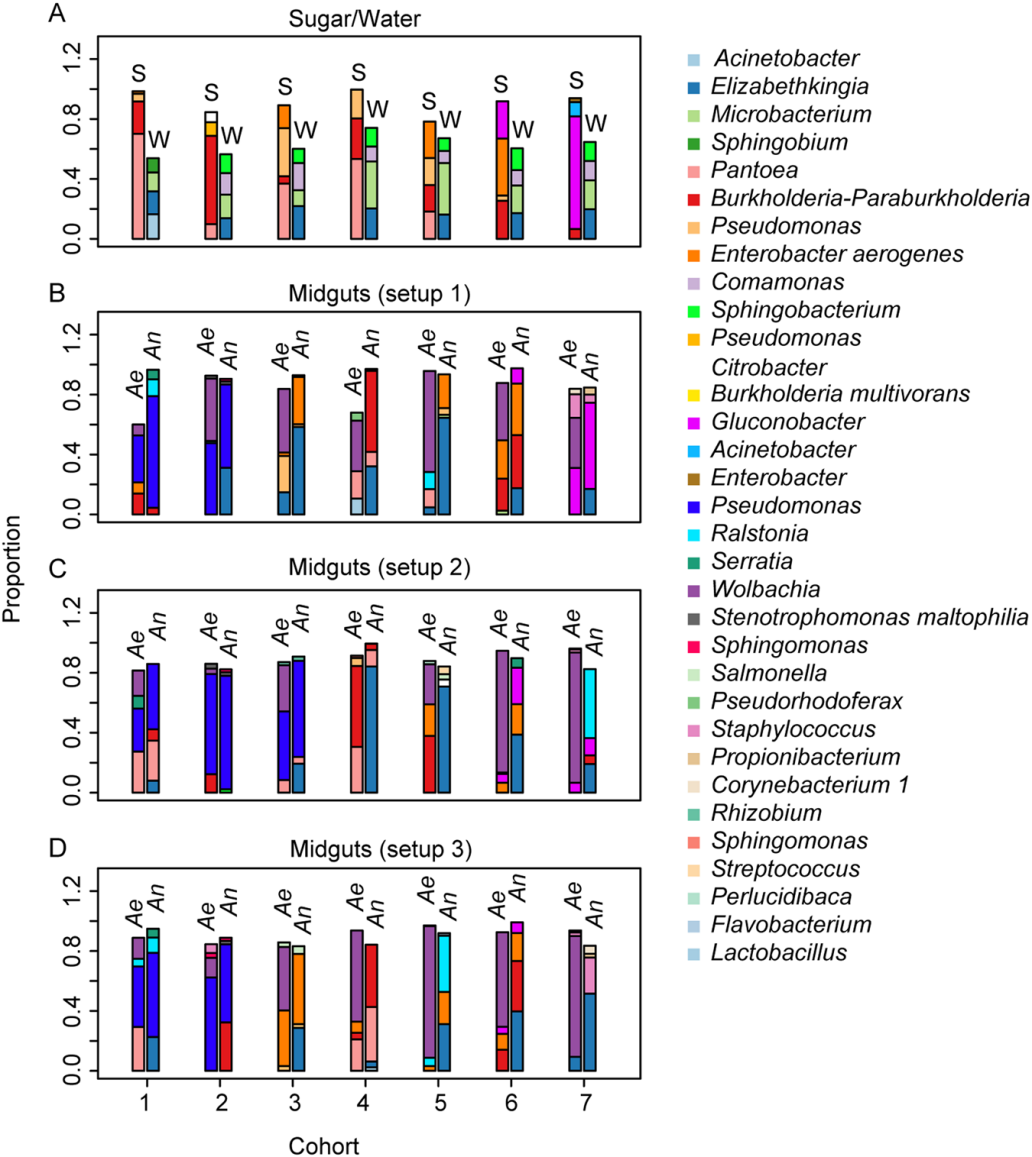
Mean proportion of the four most abundant OTUs per cohort. The two bars per cohort show the proportions in sugars and water (**A**), and in the guts of *Anopheles* and *Aedes* cultured as in setup 1 (**B**), setup 2 (**C**), and setup 3 (**D**). OTUs with a sequence similarity above 95% and correlation in abundance values above 0.9 were collapsed into a single OTU. S, sugar; W, water; Ae, *Aedes albopictus*; An, *Anopheles gambiae*.

Two mother colonies of *An. gambiae* and *Ae. albopictus* were reared separately over a period of eight months under the same conditions. From these mother colonies, seven cohorts of eggs were collected at seven different time points between the months of October and May. For each cohort, three experimental rearing setups were established (Fig. 1): setup 1, in which the two mosquito species were reared independently in separate larval pans and in separate cages; setup 2, in which the larvae of both species were co-reared in the same pan (yet separated by a net to avoid predation), but emerging adults were reared in separate cages, to analyze the effect of sharing the same larval water on the midgut microbiota of adult female mosquitoes; setup 3, whereby the two species were reared independently from each other as larvae and adults, but adult female mosquitoes of both species shared the same sugar source in order to analyze the effect of sharing the sugar source on their midgut microbiota. We determined for each combination of cohort and sample type (sugar, larval water, or midguts) the four most abundant OTUs. They were more stable across cohorts in the larval waters as compared to sugar pads, and to *Anopheles* and *Aedes* midguts (Fig. 2A). In general, *Elizabethkingia, Sphingobacterium, Comamonas* and *Microbacterium* were the four most abundant genera in the larval water in all seven cohorts (Fig. 2A). Although the *Burkholderia-Paraburkholderia* genus is dominant in the sugar pads in all cohorts, *Pseudomonas, Pantoea* and *Enterobacter aerogenes* were abundant in at least four cohorts. The sugar pads showed more variability for the dominant taxa as compared to the water (Fig. 2A). The most abundant OTUs in the *Anopheles* and *Aedes* midguts in the three different culture setups (Fig. 2B–D) were highly variable with *Elizabethkingia* and *Wolbachia* being the most dominant across most cohorts in the *Anopheles* midguts and *Aedes* midguts, respectively.

Most of the OTUs were shared between the waters of both species. This was similar for the sugar (Supplementary Fig. S1). With respect to the midgut microbiota, both species shared a large number of OTUs (540) yet 168 and 251 OTUs appeared uniquely in *An. gambiae* and *Ae. albopictus*, respectively.

A multivariate analysis of OTU abundance was used to assess the influence of food source, species and cohort on midgut microbiota-composition of the lab-reared mosquitoes. Our results revealed that the microbial composition of the midguts of all female mosquitoes was significantly associated with all predictor variables tested, namely larval water, sugar fed to adults, cohort number, and mosquito species (Table 1; *P* = 0.001 for all variables, non-parametric MANOVA). The significant effect of larval water and sugars implies that mosquitoes sharing the same food source (sugar or larval water) pooled from all cohorts exhibited a more similar microbiota composition than mosquitoes reared on independent food sources (Table 1). The significant species effect indicates that the microbial compositions of mosquito midguts belonging to the same mosquito species were more similar to each other than to those from different species. Also, within the same species, individuals belonging to the same cohort had more similar gut microbiota than those of different cohorts (*P* = 0.001, non-parametric MANOVA; Supplementary Table S2).

**Table 1:** Effect of predictors on OTU composition in mosquito midguts according to a non-parametric MANOVA.

Distance	Predictor	P-value
Bray-Curtis & altGower	Species	0.001***
	Cohort	0.001***
	Water	0.001***
Bray-Curtis	Sugar	0.001***
altGower	Sugar	0.022*

**Figure 3:**
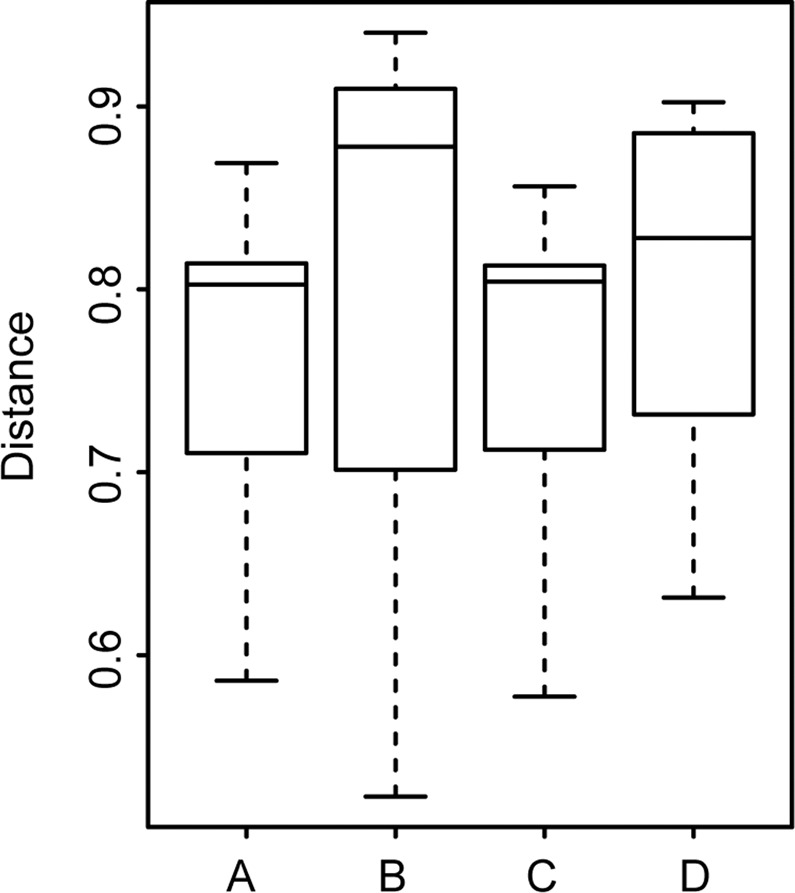
Boxplots showing the mean distances in midgut microbial composition between the two different mosquito species sharing the same environmental conditions or the same species reared in different environmental conditions. The average distance boxplots arranged in the following order: A = within species between water, B = between species within water, C = within species between sugars, D = between species within sugars. No pairwise comparison showed a significant difference (t-test).

The means of the pairwise distance between midgut microbiota of mosquitoes of different species sharing the same food source did not significantly differ from the means of the pairwise distance between the midgut microbiota of mosquitoes of the same species, but feeding on an independent food source (*P* = 0.62 for the larval water and *P* = 0.45 for sugar, t-test). In other words, food source and host species affect the midgut microbiota at roughly equal extent. This was true for both larval water and sugar (Fig. 3).

### The effect of mosquito species and cohort on the microbial compositions of larval water and sugar

We used a non-parametric MANOVA to assess the effect of the cohort and mosquito species on the microbial composition of the larval water and sugar pads. The microbial composition of both the larval water and sugar pads were significantly affected by cohort (*P* = 0.003 for larval water, *P* = 0.001 for sugars, non-parametric MANOVA; Supplementary Tables S3, S4). The mosquito species also influenced the microbial composition of the larval water (*P* = 0.001 for *An. gambiae*, *P* = 0.017 for *Ae. albopictus*, non-parametric MANOVA; Supplementary Table S3); however, a similar effect was not observed for sugar pads (*P* = 0.92 for *An. gambiae*, *P* = 0.88 for *Ae. albopictus*, non-parametric MANOVA; Supplementary Table S4). The latter result suggests that inoculation of sugar pads by mosquito activities is not a major contributor to the microbial composition in the sugar pads.

### The effect of cohort, food source and species on the midgut abundance of individual OTUs

**Figure 4:**
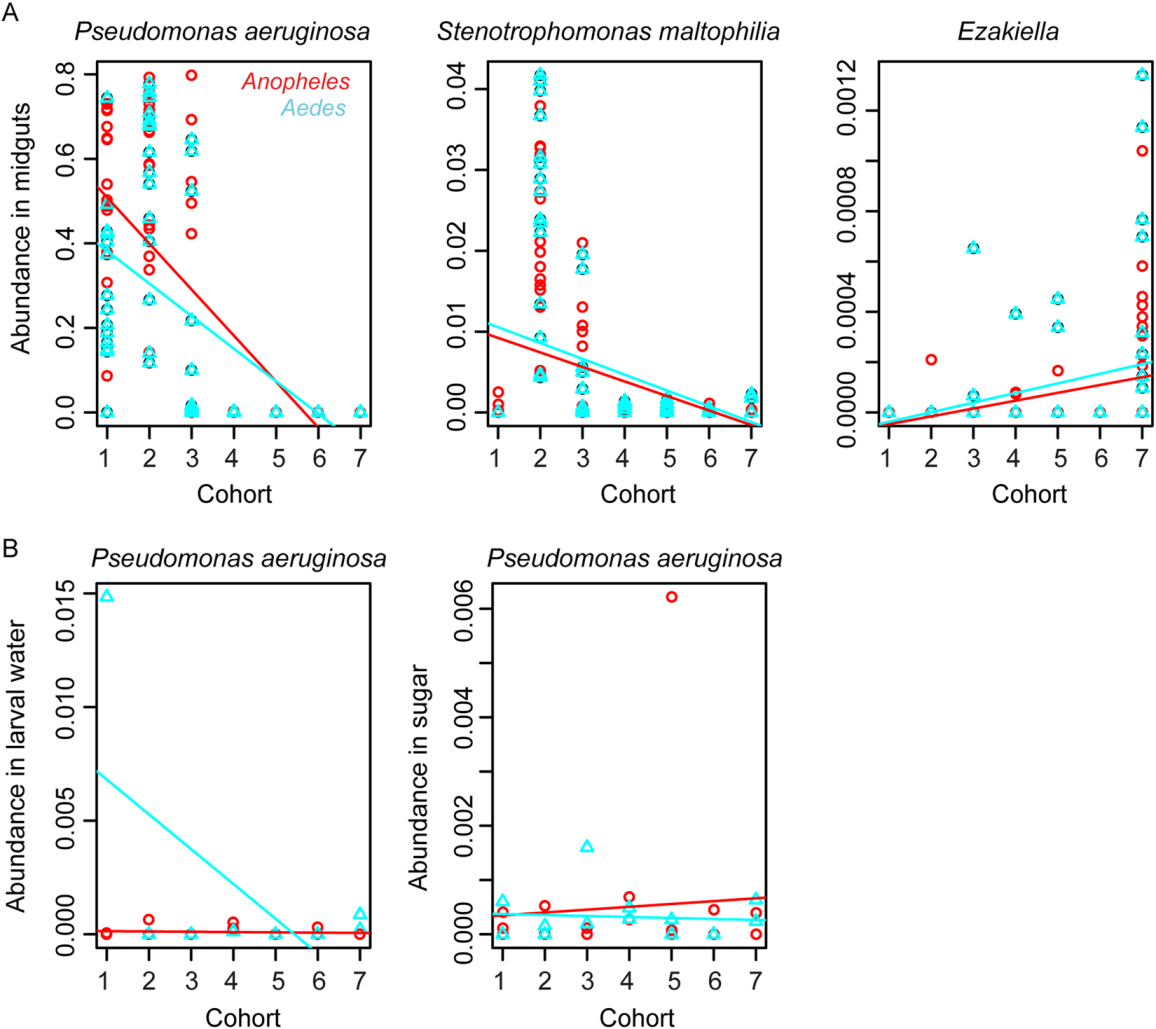
Abundance of representative OTUs across the seven cohorts. (**A**) Representative OTUs in *Anopheles* and *Aedes* midguts; in all plots, the slope differs significantly from zero with a false discovery rate of 5%. (**B**) Abundance of *Pseudomonas aeruginosa* in larval water and sugar pads. *Aedes albopictus* (blue); *Anopheles gambiae* (red).

A univariate analysis of OTU abundance was used to assess the effect of cohort, food source and species on the abundance of individual OTUs in the midguts of both adult mosquito species. This analysis showed a significant abundance trend across the seven different cohorts for 30 OTUs from 23 genera (Supplementary Table S5). For instance, *Pseudomonas aeruginosa* and *Stenotrophomonas maltophilia* are mainly detected in the first three cohorts, whereas *Ezakiella* is more abundant in the later cohorts (Fig. 4A). Interestingly, the abundance of *Pseudomonas aeruginosa* in midguts that ranges between 0% and 80% is neither reflected in larval water nor sugar (Fig. 4B), suggesting the involvement of extra-environmental factors. Furthermore, the gut abundance of 25 of the 30 OTUs that showed a significant trend across cohorts did not significantly co-vary with the abundance in water or sugar (Supplementary Table S5).

**Figure 5:**
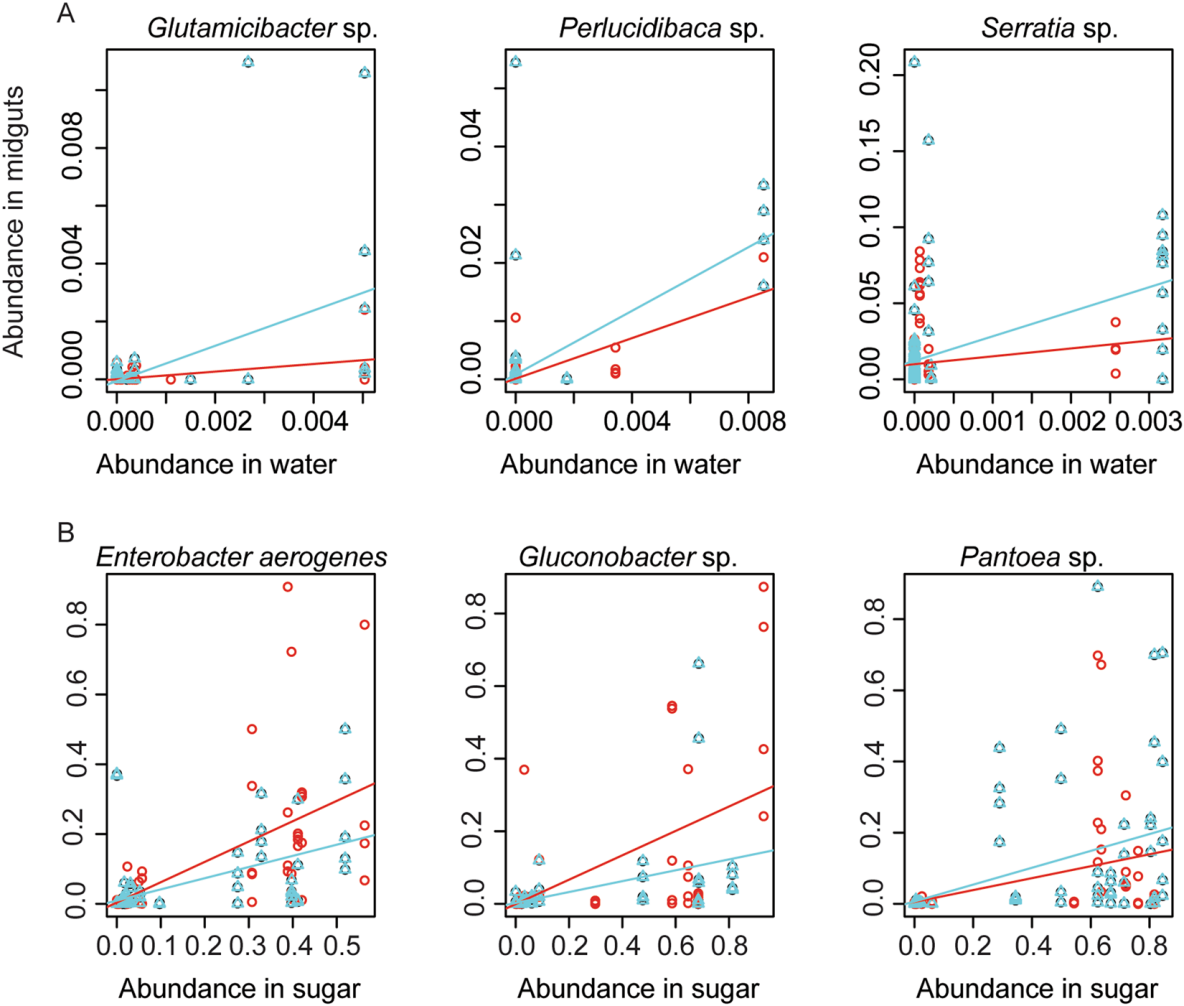
Abundance in midgut samples of representative OTUs that are significantly influenced by (**A**) abundance of OTUs in the larval water and (**B**) abundance of OTUs in sugar pads. In all plots, the slope differs significantly from zero with a false discovery rate of 5%. *Aedes albopictus* (blue), *Anopheles gambiae* (red).

The abundance of 18 OTUs significantly co-varied between mosquito midguts and larval water (Fig. 5A; Supplementary Table S5), which further confirm our previous observation that microbial compositions of midguts are affected by the larval water (Table 1). In eight out of these 18 OTUs, the relationship between abundance in the larval water and in the midguts differed significantly by mosquito species. When comparing mosquito midguts to adult-fed sugars, we also detected 18 OTUs (whereof 15 differed from those that co-varied with the larval water) whose abundance significantly co-varied between midguts and sugars (Fig. 5B; Supplementary Table S5); for eight of these OTUs the relationship between abundance in the sugars and in the midguts differed significantly by mosquito species (Supplementary Table S5).

**Figure 6:**
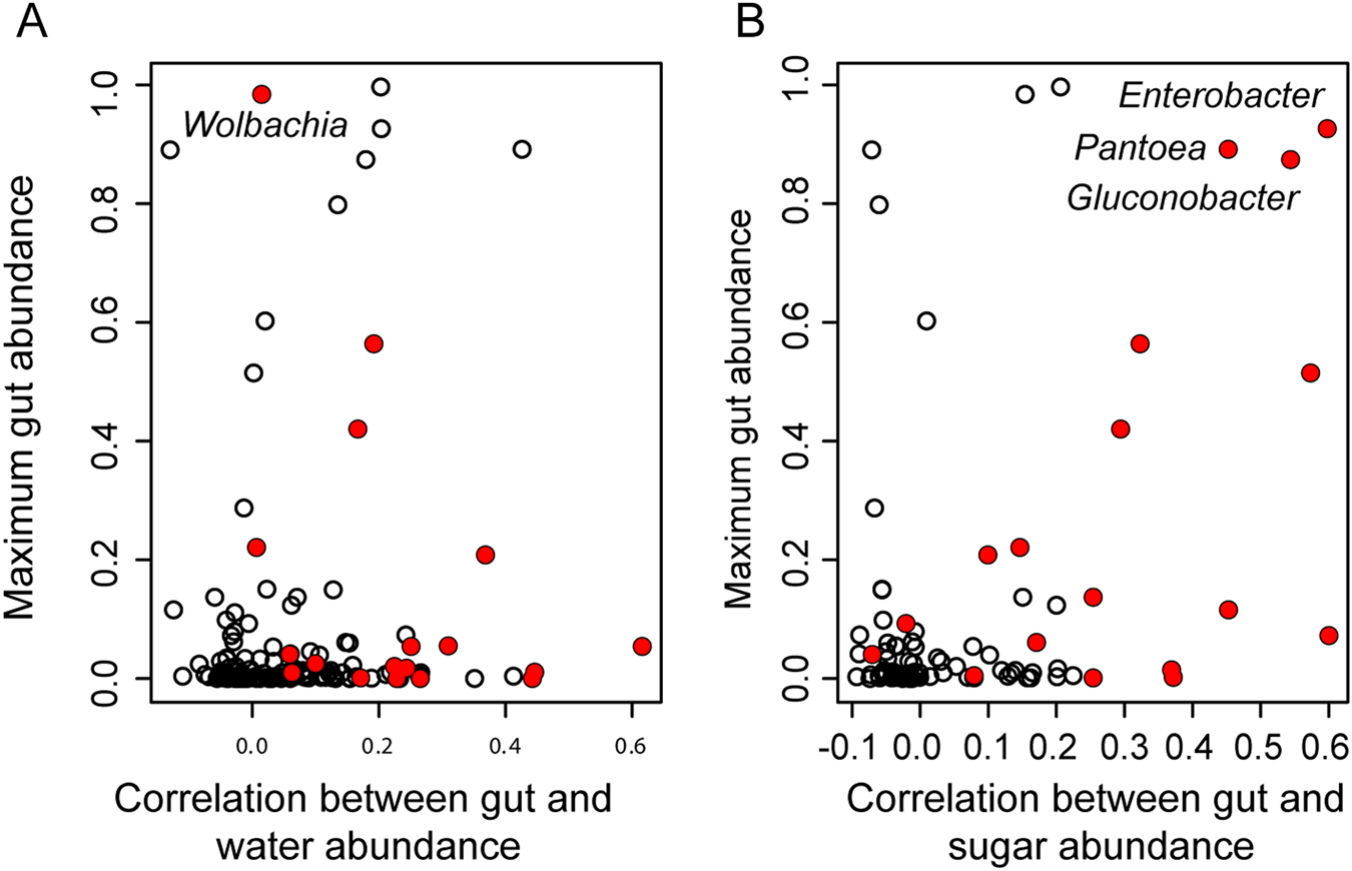
Each OTU’s maximum abundance in the midguts plotted against the correlation between (**A**) midgut and larval water abundance, and (**B**) between midgut and sugar abundance. Red circles indicate OTUs where water or sugars are a significant predictor of midgut abundance in both mosquito species with a false discovery rate of 5%. OTU genera are written next to circles corresponding to OTUs that reach a maximum abundance above 70% in midguts and whose abundance in midguts is significantly predicted by abundance in sugar.

The most abundant OTUs in the larval water are more stable across the seven different cohorts than the most abundant OTUs in sugar (Fig. 2A). However, the four most abundant OTUs in the larval water make up a smaller percentage of all OTUs in the larval water samples as compared to those in the sugar or midgut samples (Fig. 2A). An OTU’s maximum abundance in midguts is significantly correlated with its correlation between water and midgut abundance (*r* = 0.17, *P* = 0.02, Spearman correlation; Fig. 6A) and with its correlation between sugar and midgut abundance (*r* = 0.45, *P* = 0.03, Spearman correlation; Fig. 6B). In other words, OTUs that reach a high abundance in the midguts tend to show a similar abundance pattern between midguts and environment. Three out of the six most abundant OTUs, which constitute more than 80% of the sequence reads in any midgut from both species, show a high correlation (above 0.4) between sugar source and midgut (Figs. 5B, 6B); however, none of these six OTUs showed such a high correlation with the larval water (Fig. 6A).

The midgut abundance of two OTUs (OTU1 *Elizabethkingia* and OTU575 *Wolbachia*) differed significantly between mosquito species (Supplementary Table S5) in the univariate analysis of OTU abundance. While *Wolbachia* is an intracellular bacterium and not considered as part of the midgut microbiota, the fact it was detected in our assay is probably due to its intracellular presence in midgut epithelial cells. *Wolbachia* is known to infect several tissues in *Ae. albopictus* including the gut^25^. The midgut abundance of 14 OTUs depended on either sugar or larval water in a manner that differed between the mosquito species, i.e., 14 OTUs showed significant water by species or sugar by species interaction (Supplementary Table S5). We repeated the MANOVA excluding OTU1 and OTU575 and still found a significant effect of the mosquito species on the midgut microbiota (*P* = 0.001, non-parametric MANOVA). Hence, the MANOVA, which combines abundance values of all OTUs, could detect differences in gut microbiota between the mosquito species, even when no single OTU differed significantly between species. This apparent contradiction between MANOVA and the univariate analysis occurred either because many OTUs differed weakly between the mosquito species and did not appear as significant in the univariate analysis, or because the effects of mosquito species on OTU abundance interact with environmental effects, as shown for the 14 OTUs in our analysis. It is worth noting that the 5% false discovery rate adjustment in the univariate analysis further reduced the detection of OTUs with weak species effects. Hence, despite that the Venn diagrams of OTU overlap between the midguts of both species identified several species-specific unique OTUs (Supplementary Fig. S1), almost all of these seem to differ weakly between both species. This resulted in only *Elizabethkingia* and *Wolbachia* showing significant changes in abundance in our univariate analysis.

The percentage abundance of *Elizabethkingia* in adult mosquito midguts from co-cultured larval settings further emphasizes the species effect. Despite its high prevalence in larval water, the percentage of *Elizabethkingia* in co-reared mosquitoes of each batch is 8- to 1169-fold higher in the midguts of *Anopheles* compared to *Aedes* (Table 2). Additionally, *Burkholderia* was one of the OTUs constituting more than 90% of the reads in the sugar samples shared by both adult mosquito species. When the abundance of *Burkholderia* in sugar was greater than 6%, the number of reads in *Anopheles* mosquitoes midguts was 2 to 36-fold that in *Aedes* (Table 2); although this observed difference between both species was not statistically significant, this trend was observed in 4 out of the 6 cohorts analyzed.

**Table 2:** Percentage of reads found in co-cultured mosquitoes and in larval water and sugar, respectively.

Batch	Elizabethkingia		Burkholderia-Paraburkholderia	
	Ae/An^a^	Water^b^	Ae/An^a^	Sugar^b^
1	0.19/7.99	12.24	2.61/0.32	6.58
2	0.13/1.03	3.27	0.91/32.31	79.84
3	0.10/19.28	7.31	—	—
4	0.10/84.25	20.88	4.36/41.57	28.82
5	0.06/70.80	3.89	0.25/1.64	11.99
6	0.14/38.69	3.18	14.11/33.65	30.36
7	0.07/19.07	17.76	0.01/0.03	0

OTUs with a sequence similarity above 95% and correlation in abundance values above 0.9 were collapsed into a single OTU.

^a^Average of 3–5 samples per batch.

^b^One sample per batch.

## Discussion

The gut microbial composition is complex and depends on several factors such as the acquisition of environmental microbes, the gut physiological niche favoring certain types of microbes and microbial interactions in the gut. Although the outside environment is known to strongly influence the gut microbiota associated with mosquitoes^8,12,20^, different mosquito species collected from the same locations still show clear differences in their gut microbiota^10,11^, which could imply species differences in feeding behavior and/or physiology. In this study, we wanted to explore the impact of species differences on the gut microbiota of mosquitoes sharing the same environment. A previous study by Coon *et al*. (2014) compared the microbiota in mosquitoes of three different species physically separated in the same laboratory environment, but exposed to the same conditions including food, and found that the mosquitoes still developed a species-specific microbiota with between 26% and 48% of the OTUs being unique for each species. We wanted to push the comparison between species to the extreme by co-rearing two mosquito species of different subfamilies in the same environment (i.e. sharing the larval water) with access to the very same food source, but still physically separated. Thus, as far as possible negate the environmental differences in the culture conditions of both species.

Seven cohorts of mosquitoes were taken from their mother colonies of *Anopheles gambiae* and *Aedes albopictus* over a period of seven months. Thus, although not being true generations, i.e., that one cohort gives rise to the next, the cohorts were formed in a sequential manner and influenced by the environmental changes in the insectary. In our study, 30 OTUs showed a significant abundance trend over the cohorts, but only five of these were also correlated to the abundance in larval water or sugar. The significant **effect of cohort** implies that the microbial compositions of mosquito midguts belonging to the same cohort were more similar to each other than to those of a different cohort; this effect may be attributed to changes in the microenvironment of the insectary between the different cohort collection times or to genetic changes in the mother colonies. If the OTU abundance trends were due to genetic changes, such changes must have occurred due to differential fertility since we did not observe any substantial mortality. Changes in the microenvironment of the insectary may be attributed to environmental microbes brought in by human activity or through air. The fact that the seven cohorts were collected between the months of October and May, extending over three seasons, fall, winter and spring, suggests that between the different collection dates, the environmental changes that would impact the types of microbes brought into the insectary can be significant. A similar observation was made by Coon *et al*.^26^, who reported that seasonal changes did influence the bacterial communities in larvae and their aquatic habitats in the field. We also found that the environmental fluctuations are not mediated in a straight-forward way through either water or sugar. For example, the striking change across cohorts of the abundance of *Pseudomonas aeruginosa* in midguts that ranges between 0% and 80% is neither reflected in the abundance in larval water nor sugar (Fig. 4). Hence, for some OTUs, large changes in midgut microbiota across cohorts may be driven by complex bacterial interactions within the mosquito midguts. These large variations in the abundance of certain OTUs between individuals of the same species are not uncommon according to previous reports^8,11^. *Pseudomonas* has previously been shown to vary substantially in abundance between individuals; for example, it was shown to constitute more than 80% of midgut OTUs in certain individuals of field caught *Aedes aegypti*, *Anopheles funestus* and *Mansonia africana*^11^ and more than 70% in *Ae. albopictus*^27^. It remains unclear what drives these inter-individual fluctuations of certain OTUs.

We analyzed further the impact of the environment on the midgut microbiota of adults. We found a significant **effect of larval water and sugars**, which implies that mosquitoes sharing the same food source (sugar or larval water) pooled from all cohorts exhibited a more similar microbiota composition than mosquitoes reared on independent food sources, suggesting that despite being in the same physical space (i.e. the same insectary room), individual larval water pans and adult sugar pads may develop distinct microenvironments. It is worth noting that differences in the microbial contents of larval waters may be transstadially transmitted to adult midguts^4,19^, or directly when adult mosquitoes imbibe larval water during emergence^18^. We also observed the reverse effect, i.e., that mosquitoes affect the microbial composition of their larval food source. The species effect on the microbial composition in the larval water is most likely driven by excretions of the larval stages and possibly reflects the effects of the differences in the gut microenvironments of both larval species (although we did not sequence the larval gut microbiota to confirm this observation). Inoculation of larval habitats by bacteria selected for in the larval midguts has been also proposed by Coon *et al*.^26^. The data also suggest that the most abundant OTUs in the adult midguts are more closely correlated with microbiota in sugars than in the larval water. The microbial composition of the sugar was not shown to be influenced by the mosquito species, and therefore it can be inferred that the correlation between abundance of specific OTUs in sugars and in midguts are due to the microbiota in the sugars influencing that of the midguts and not *vice versa*. None of the major OTUs present in the larval water appear as major OTUs in the sugar, suggesting that the transfer of bacteria between larval water and sugars by newly emerging adult mosquitoes (through imbibing or direct contact with water) is unlikely to contribute significantly to the microbial composition of the sugar. This is also supported by the fact that the four major OTUs in the water are more stable across cohorts as compared to those in the sugar pads, which exhibit more variations. These variations are likely to be imposed by the environment and the chemical characteristic of the food source medium.

Our data also showed that the microbial composition of mosquito midguts belonging to the same mosquito species were more similar to each other than to those from different species in the pooled cohorts. The observed **species effect** may be due to different physiological responses in the midguts of both species. It may also reflect the different feeding behaviors of the larvae of both species. *An. gambiae* larvae feed preferentially on surface microlayers^17,28^ while larvae of *Aedes* spp. browse for food in a water column^17^ with *Ae*. *albopictus* foraging mainly in the middle or bottom of their containers^29^. While these different feeding modes might directly influence the larval gut microbiota (which was not the focus of this work), they could also indirectly influence the gut microbiota of adults through transstadial transmission^4,19^. It is worth noting here that for both *Aedes* and *Anopheles* larval cultures, water was collected at a depth roughly half-way between the water surface and the bottom of the pan (the depth of water in our pans being approximately 1  cm). Since bacterial communities differ between the surface and bottom layers of water^12^, our collection mode of larval water may not represent all microecological niches present in the larval pans. Nevertheless, we believe that the larval water microbiota in our samples still represents relatively well the total bacterial communities in the pans for two reasons: First, the water depth in our pans is small. Second, the pans were moved from the incubator to the working bench before water was collected which results in significant mixing of water layers. Altogether, our data reveals that any OTU abundance correlations between larval water and mosquito midguts can be due to bi-directional effects whereas OTU abundance correlations between sugar and midguts are most likely due to mosquitoes ingesting microbes present in the sugar.

Moreover, mosquito larvae sharing water pans may also experience intraspecific competition. Although it remains unclear how this competition may influence the midgut microbiota of adult mosquitoes, it is known to affect development time, larval survival and size, and fitness of emerging adults as shown for several mosquito species including *An. gambiae*^30–33^ and *Ae. albopictus*^34–36^. However, in our colonies we expect minimal intraspecific competition since larval pans are always maintained at low densities (approximately 100–120 larvae/pan with an area of 768  cm^2^) and there is no shortage of food; we also did not notice any irregularities neither in the size of same-age larvae nor in the developmental times for single-species cultures (although this data was not recorded). However, in our mixed-species populations (i.e. larval co-culture), we noticed that *An. gambiae* larvae co-cultured with *Aedes* required longer time to pupate as compared to those in single-species cultures (although we did not record the time for pupation). Interestingly, these data suggest that despite the physical barrier separating both larval species, the mere presence of *Ae. albopictus* in the same pan as *An. gambiae* affects larval development of the latter. In fact, the superiority of *Ae*. *albopictus* as a resource competitor with respect to other mosquito species has been previously noted^34,37,38^.

Our univariate analysis identified two OTUs (*Elizabethkingia* and *Wolbachia*) that significantly differed in abundance between *An. gambiae* and *Ae. albopictus* in the pooled cohorts. *Wolbachia* is an intracellular bacterium that is vertically transmitted and is not considered part of the gut microbiota; it was detected in our study likely because of its ability to infect the midgut epithelial cells in *Ae. albopictus*^25^. This leaves *Elizabethkingia* as the sole OTU showing significantly distinct abundance between both species. By excluding these two OTUs, a MANOVA still detected a significant species-effect on the midgut microbiota suggesting that certain OTUs differed weakly between the mosquito species and did not appear as significant in the univariate analysis. The fact that no other OTU showed significant species-specific abundance may be due to the insectary environment imposing a dominant and selective effect on microbiota composition. In support of this, a recent study showed that when geographically diverse colonies of *Ae. aegypti* were reared in the same insectary they harbored the same adult midgut microbiota^16^. The difference in colonization efficiency of certain bacterial species may reflect specific interactions with other gut microbes as well as with the physiological responses of the host that might differ between both species and between individuals of the same species due to gene polymorphisms. For instance, data from *An. gambiae* show that natural genetic variation in immune-related genes shape the gut microbiota with high specificity^39^. In *Aedes aegypti*, recent data suggest instead that the natural genetic variation in amino acid metabolism influences the adult midgut bacterial load of individual female mosquitoes^40^.

Altogether, our data support the conclusion that the mosquito midgut microbiota is affected by food source, cohort and species. Even though the microbiota differs significantly between the mosquito species, considerable intraspecies variations in midgut microbiota between cohorts indicate that it is difficult to define a core microbiota in mosquitoes. Our study has shown that the situation is even more complex. The midgut abundance of multiple OTUs showed striking changes across different cohorts that were consistent between mosquito species, but were not reflected in larval water or sugars. Hence, in addition to the environmental variations and species effect, the gut microbiota is also modulated by complex microbial interactions.

## Methods

### Ethical statement

This study was carried out according to the recommendations in the Guide for the Care and Use of Laboratory Animals of the National Institutes of Health (Bethesda, USA). The animal protocol was approved by the Institutional Animal Care and Use Committee IACUC of the American University of Beirut (permit number 16-03-369). The IACUC functions in compliance with the Public Health Service Policy on the Humane Care and Use of Laboratory Animals (USA), and adopts the Guide for the Care and Use of Laboratory Animals of the National Institutes of Health.

### *Anopheles gambiae* and *Aedes albopictus* rearing

Experiments were done using *Anopheles gambiae* G3 strain and *Aedes albopictus* SARBA strain [isolated from Lebanon^41^]. Both mosquito species were reared in the same insectary and conditions (including diet) and maintained at 27 °C (± 0.5) and 80% (± 5%) humidity with a 12  h day-night cycle. Larvae of both species were reared in autoclaved distilled water at low densities of approximately 100–120 larvae/pan of area 768  cm^2^ in order to avoid competition. Larvae were fed on TetraMin tropical fish food which was grinded into fine particles, but not sterilized, while adults were given sugar pads containing 10% sucrose that was sterilized by autoclaving. Adult females of both mosquito species were given BALB/c mice blood (mice were anaesthetized with ketamine) once per week for egg production. From these mother colonies of *An. gambiae* and *Ae. albopictus,* eggs were collected at seven different time points between the months of October and May to establish the experimental culture setups detailed in the section below. Each of these egg collections is considered as one cohort.

### Experimental design for studying the dynamics of mosquito gut microbiota

The first setup (Fig. 1A) was prepared to analyze how stable the midgut microbiota structure is in the two different lab-reared mosquito species across the seven cohorts when each species was reared independently in different larval pans and in separate cages, but under the same conditions. In the second setup (Fig. 1B), the two species were co-cultured in the same pan to analyze the effect of sharing the same larval water (which contain larval food) on adult midgut microbiota. However, the different mosquito species were separated by a net to avoid predation by *Aedes* larvae on those of *Anopheles*. Pupae from the co-cultured species were collected at the same time into different cages and adults were fed on independent sugar pads. The third setup (Fig. 1C) was prepared to analyze the effect of sharing the sugar source on adult midgut microbiota. The two species were reared independently from each other as larvae and adults, but adults of both species shared the same sugar source (a cotton pad containing 10% sucrose solution). In the three setups, adults of both species were reared in separate cages containing approximately 30 mosquitoes each. In all these setups, four-days-post-emergence adult female mosquitoes were anesthetized on ice and their midguts were dissected in sterile PBS with clean forceps (sterilized by dipping them in 70% ethanol between different dissections) and placed in sterile eppendorf tubes. Although we did not surface sterilize mosquitoes before dissection, all midguts were washed with sterile PBS (1×x) after transfer to Eppendorf tubes to eliminate any bacteria carried over during the dissection procedure. We dissected 10 midguts (the hind and foregut were removed) per experimental sample (as defined in Fig. 1) per cohort, however only 4 to 5 midguts were sequenced per sample, chosen based on the amount of DNA recovered after purification. Midguts were individually frozen at −20 °C and processed later for bacterial DNA extraction. During the course of this experiment, samples were collected systematically from the sugar pads and larval waters to analyze their bacterial content in order to assess the impact of the microbiota of the larval water and the sugar pads on the structure of the mosquito gut microbiota. Larval waters were collected on the day of pupation; 1  ml volumes were collected from each pan from beneath the surface layers without touching the bottom of the pan. As to sugar samples, 1  ml volume of the sugar solution was collected on day 4 post-adult emergence (i.e. on the same day adult midguts were dissected) by squeezing the sugar cotton pads into sterile eppendorf tubes. Tubes containing solutions from sugar and larval water were centrifuged at 4000 g for 5  min and bacterial pellets were stored at −20 °C until DNA extraction.

### DNA extraction, PCR amplification and barcoding

DNA was extracted from mosquito midguts and pellets, obtained from the sugar and larval water, using the Qiagen blood and tissue kit according to the manufacturer’s instructions. The universal degenerated bacterial primers 341 F, 5′-CCTACGGGNGGCWGCAG-3′ and 805 R, 5′-GACTACHVGGGTATCTAATCC-3′ were used to amplify the bacterial 16S rRNA gene in the first of two PCRs using illustra™ puRe Taq Ready-To-Go™ PCR Beads (GE Health Care). Each sample was amplified individually starting by a denaturation step at 95 °C for 5  min followed by 30 cycles of [95 °C for 40  sec, 53 °C for 40  sec and 72 °C for 1  min] and ending with an extension step at 72 °C for 7  min as described^42^. The second PCR was performed following the same conditions as the first PCR, but only for 10 cycles of iteration in the presence of 1 out of 50 barcoded primer pairs, as described^42^. This was done in order to make one sequence library for 50 samples as one sample and later be able to differentiate between them. Appropriate negative controls were included in the PCR reaction to ensure the absence of bacterial DNA contamination independent of the experimental design. No technical replicates were performed on samples.

### Library preparation and sequencing

Libraries were prepared from 10  ng of amplicon sample using the ThruPLEX DNA-seq Prep Kit (Rubicon Genomics) according to the manufacturer’s instructions. The quality of the libraries was evaluated using the TapeStation from Agilent Technologies using the D1000 ScreenTape. The adapter-ligated fragments were quantified by qPCR using the Library quantification kit for Illumina (KAPA Biosystems) on a StepOnePlus instrument (Applied Biosystems/Life technologies) prior to cluster cohort and sequencing. Sequencing was performed by the SNP&SEQ Technology Platform, a national facility within the National Genomics Infrastructure (NGI), hosted by Science for Life Laboratory, in Uppsala, Sweden (https://www.scilifelab.se/facilities/snpseq/). Sequencing was carried out on Illumina MiSeq instrument (MCS v 2.6.2.1/ RTA v1.18.54) according to the manufacturer’s instructions. Demultiplexing and conversion to FASTQ format was performed using the bcl2fastq2 software (v2.19.0.316), provided by Illumina (http://support.illumina.com/sequencing/sequencing_software/bcl2fastq-conversion-software.html). Additional statistics on sequencing quality were compiled with an in-house script from the FASTQ-files, RTA and bcl2fastq2 output files. All reads from this study are available at the European Nucleotide Archive under accession number PRJEB28193.

### Processing of sequences

Using the program Mothur^43^ (https://www.mothur.org), forward and reverse reads generated by MiSeq were merged and barcodes and primers were removed. The merged reads were quality filtered to remove reads longer than 534  bp and shorter than 394  bp. Reads with ambiguous bases were removed and also those with homopolymers of more than 8. In VSEARCH (https://github.com/torognes/vsearch) the reads were dereplicated and singletons removed^44^. Chimeras were removed using the UCHIME algorithm^45^. *De novo* clustering after chimera detection was done at 97% similarity. Finally, all reads before dereplication were mapped to the representative sequences at 97% similarity threshold to generate the OTU table. In QIIME^46^ (http://qiime.org), taxonomy was assigned using the classification software UCLUST^47^ with reference database and taxonomy from SILVA 128 release^48,49^ clustered at 97% similarity. Some OTUs were defined based on DNA sequences that did not belong to 16S rDNA. These OTUs were identified by aligning all sequences representative for an OTU to the known *Elizabethkingia* 16S rRNA gene using the function *pairwiseAlignment* from the Bioconductor R package *Biostrings*. All of the alignments that gave an alignment score lower than −500 were considered non-bacterial sequences and the corresponding OTUs were removed from the analysis.

### Multivariate analysis of OTU abundance

All analyses were performed on standardized OTU abundance values. Standardization was applied by dividing the OTU abundances by the sum of OTU abundances per sample. Effects of different predictor variables on the microbial composition of samples were assessed using a non-parametric MANOVA that analyzes distances in a multidimensional space defined by OTU abundances. All analyses were repeated for two different distance measures, the modified Gower distance (*altGower*) and *Bray-Curtis* distance^50^. A non-parametric MANOVA was performed to test for effects of mosquito species, cohort, larval water, and sugars fed to adults on the midgut microbiota composition. All non-parametric MANOVAs in this study were done using the *adonis* function from the R package *vegan*^51^. Since none of the conclusions depended on the distance measure, we report in the main text only the results obtained based on the *Bray-Curtis* distance.

A separate analysis was conducted to directly compare the influence of mosquito species with the influence of food source (sugar or larval water) on the mosquito midgut microbiota. To compare the relative influence of mosquito species and larval water, two different mean distances were calculated per cohort. The first mean distance was calculated among all pairwise distances between samples from two mosquitoes that were co-cultured in the same larval pan, but belonged to different species. The second mean distance was calculated among all pairwise distances between samples from two mosquitoes of the same species that were reared in different larval pans. This procedure yielded two mean distances per cohort. After testing for normality using the Shapiro-Wilk test (R function *shapiro.test*), a Student’s t-test (R function *t.test*) was used to test whether there was a difference between these two sets of mean distances. An entirely analogous method was applied to compare the effect of mosquito species and sugar on the mosquito midgut microbiota.

Two separate non-parametric MANOVAs were performed to test for effects of the mosquito species and cohort on the microbial composition of the larval water and sugars. In both analyses, mosquito species and cohort were the predictor variables, but one analysis was performed on all microbial samples from larval water and the other on all sugar samples.

### Univariate analysis of OTU abundance

The effects of mosquito species, cohort, OTU abundance in larval water and OTU abundance in sugar on the standardized OTU abundance in midgut samples were tested separately for each OTU using a linear model (R function *lm*). A false-discovery rate of each effect was estimated using the Benjamini-Hochberg correction^52^. Moreover, two correlations were calculated for each OTU. One correlation was between the abundance of an OTU in the larval water samples and the abundance of the same OTU in the midguts of mosquitoes that were reared in the respective larval water. The other correlation was between the abundance of an OTU in the sugar samples and the abundance of the same OTU in the midguts of mosquitoes that were fed on the respective sugar. Each of these two sets of correlations was tested for a correlation with an OTU’s maximum abundance in midgut samples using the *spearman_test* function from the R package coin.

## Supplementary information

Supplementary information.

## Data Availability

All sequence reads from this study are available at the European Nucleotide Archive under accession number PRJEB28193.
