# Supplementary material for: The environment and species affect gut bacteria composition in laboratory co-cultured Anopheles gambiae and Aedes albopictus mosquitoes
Source: Sci Rep. 2020 Feb 25;10:3352. doi: 10.1038/s41598-020-60075-6 (PMC7042291; doi:10.1038/s41598-020-60075-6)

**Supplementary information**

**The environment and species affect gut bacteria composition in laboratory co-cultured *Anopheles gambiae* and *Aedes albopictus* mosquitoes**

Sally A. Saab^1^, Heinrich zu Dohna^1^, Louise K. J. Nilsson^2,3^, Piero Onorati^2^, Johnny Nakhleh^1^, Olle Terenius^2,3*^, Mike A. Osta^1*^.

^1^ Department of Biology, American University of Beirut, Bliss Street, Beirut, Lebanon

^2^ Department of Ecology, Swedish University of Agricultural Sciences (SLU), Box 7044, SE-750 07 Uppsala, Sweden

^3^ Department of Cell and Molecular Biology, Microbiology, Uppsala University, BMC, Box 596, SE-75124, Uppsala, Sweden.

* Corresponding authors

**Running title**: Microbiota dynamics in co-reared mosquito species

Table S1

Table S2

Table S3

Table S4

Table S5

Figure S1

| **Table S1:** Mean abundance of bacterial phyla in larval waters, sugar pads, *Anopheles* and *Aedes* midguts | | | | | |
| --- | --- | --- | --- | --- | --- |
|  |  | **Mean abundance** | | | |
| **Phylum** | **OTUs per Phylum** | **Water** | **Sugar** | ***An. gambiae*** | ***Ae. albopictus*** |
| *Bacteroidetes* | 128 | 0.41 | 0.0007 | 0.3 | 0.03 |
| *Proteobacteria* | 318 | 0.35 | 0.99 | 0.63 | 0.9 |
| *Firmicutes* | 161 | 0.004 | 0.001 | 0.03 | 0.03 |
| *Spirochaetae* | 8 | 0 | 0 | 6.02* 10 ^-5^ | 0.0001 |
| *Actinobacteria* | 120 | 0.21 | 0.0001 | 0.02 | 0.02 |
| *Verrucomicrobia* | 6 | 0.007 | 0 | 4.63*10 ^-5^ | 1.3*10 ^-5^ |
| *Unassigned* | 41 | 0 | 0 | 7.21*10 ^-5^ | 0.0002 |
| *Parcubacteria* | 59 | 0 | 0 | 0.00013 | 0.0003 |
| *Acidobacteria* | 9 | 0 | 0 | 4.01*10 ^-5^ | 1.93*10 ^-5^ |
| *Saccharibacteria* | 32 | 0 | 0 | 0.0002 | 0.0003 |
| *Fusobacteria* | 11 | 1.52*10^-5^ | 0 | 0.0007 | 0.0007 |
| *Cyanobacteria* | 19 | 0.0002 | 1.14*10 ^-5^ | 0.0016 | 0.0007 |
| *Chloroflexi* | 10 | 0 | 0 | 3.77*10 ^-5^ | 6.9*10 ^-5^ |
| *Deinococcus-Thermus* | 6 | 0 | 0 | 3.76*10 ^-5^ | 2.38*10 ^-5^ |
| *Tenericutes* | 6 | 0 | 0 | 1.02*10 ^-5^ | 2.19*10 ^-5^ |
| *Planctomycetes* | 7 | 9.59*10 ^-5^ | 0 | 3.92*10 ^-6^ | 1.13*10 ^-5^ |
| *SR1 (Absconditabacteria)* | 4 | 0 | 0 | 3.07*10 ^-5^ | 2.43*10 ^-5^ |
| *Chlamydiae* | 9 | 0 | 0 | 1.59*10 ^-5^ | 1.7*10 ^-5^ |
| *Gracilibacteria* | 4 | 0 | 0 | 7.07*10 ^-6^ | 1.04*10 ^-5^ |
| *Peregrinibacteria* | 1 | 0 | 0 | 2.04*10 ^-6^ | 0 |
| *TM6 (Dependentiae)* | 1 | 0 | 0 | 2.04 *10 ^-6^ | 0 |
| *Synergistetes* | 1 | 0 | 0 | 0 | 6.45 * 10 ^-6^ |
| *Candidatus Berkelbacteria* | 2 | 0 | 0 | 4.58 * 10 ^-6^ | 9.31 * 10 ^-7^ |
| *WWE3* | 1 | 0 | 0 | 0 | 1.4 * 10 ^-6^ |
| *Elusimicrobia* | 2 | 0 | 0 | 3.25 * 10 ^-6^ | 5.47 * 10 ^-6^ |
| *Gemmatimonadetes* | 1 | 0 | 0 | 1.24 * 10 ^-5^ | 0 |

| **Table S2:** Effect of cohort on OTU composition in the midguts of the two different mosquito species according to a non-parametric MANOVA | | | |
| --- | --- | --- | --- |
| **Distance** | **Predictor** | **Species** | **P-value** |
| Bray-Curtis & altGower | Cohort | *An. gambiae or Ae. albopictus* | 0.001*** |

| **Table S3**: Effect of predictors on OTU composition in larval water according to a non-parametric MANOVA | | |
| --- | --- | --- |
| **Distance** | **Predictor** | **P-value** |
| Bray-Curtis | *An. gambiae* | 0.001*** |
|  | *Ae. albopictus* | 0.017** |
|  | Cohort | 0.003** |
| altGower | *An. gambiae* | 0.001*** |
|  | *Ae. albopictus* | 0.040* |
|  | Cohort | 0.015* |

| **Table S4**: Effect of predictors on OTU composition in sugars according to a non-parametric MANOVA | | |
| --- | --- | --- |
| **Distance** | **Predictor** | **P-value** |
| Bray-Curtis | *An. gambiae* | 0.92 |
|  | *Ae. albopictus* | 0.88 |
|  | Cohort | 0.001*** |
| altGower | *An. gambiae* | 0.84 |
|  | *Ae. albopictus* | 0.98 |
|  | Cohort | 0.003** |

| **Table S5:** Effect of the different factors on each OTU in the mosquito midgut | | | | | | |
| --- | --- | --- | --- | --- | --- | --- |
|  | Genus | Cohort | Larval  water | Larval water by species | Sugar | Sugar by species |
| *Elizabethkingia* | 0.006 |  |  |  |  |  |
| *Enterobacter aerogenes* |  |  |  |  | 0.0003 |  |
| *×Ferruginibacter* |  |  | 2*10^-9^ | 2.12*10^-7^ |  |  |
| *Geobacillus stearothermophilus* |  | 0.035 |  |  |  |  |
| *Sediminibacterium* |  |  |  |  | 2.45*10^-5^ |  |
| *×Sphingobium* |  |  |  | 0.023 |  |  |
| *Anaerococcus* |  | 0.018 |  |  |  |  |
| *Thermomonas* |  | 0.006 |  |  |  |  |
| *Chitinophagaceae* |  |  | 0.001 |  |  |  |
| *Neisseria* |  |  | 0.0009 |  |  |  |
| *Corynebacterium 1* |  | 0.036 |  |  |  |  |
| ^+^ *Pseudomonas* |  |  |  |  | 3.92*10^-17^ | 0.009 |
| *Tepidimonas* |  | 0.029 |  |  |  |  |
| ^+^ *Comamonas* |  |  |  |  | 0.014 | 0.009 |
| *Sorangium* |  | 0.03 |  |  |  |  |
| *Stenotrophomonas* |  | 0.0001 |  |  |  |  |
| *Burkholderia-Paraburkholderia* |  |  | 0.013 |  | 0.042 |  |
| *Pseudomonas* |  |  |  |  | 9.55*10^-15^ |  |
| *×Glutamicibacter* |  |  | 6.8*10^-9^ | 0.011 |  |  |
| *Varibaculum* |  | 0.0001 |  |  |  |  |
| *Pseudomonas* |  | 2.26* 10^-21^ |  |  |  |  |
| ^+^ *Serratia* |  | 0.0003 | 0.0005 |  |  | 0.015 |
| *Acidibacter* |  | 0.001 |  |  |  |  |
| ^×+^*Corynebacterium 1* |  |  |  | 2.27*10^-5^ |  | 0.0002 |
| × *Mesorhizobium* |  |  |  | 0.048 |  |  |
| *Pseudomonas aeruginosa* |  | 6.75*10^-26^ |  |  |  |  |
| ^+^ *Altererythrobacter* |  |  |  |  | 5.47*10^-13^ | 1.63*10^-12^ |
| *×Wolbachia* | 0.0009 | 0.0006 | 0.013 | 0.04 |  |  |
| *Stenotrophomonas maltophilia* |  | 0.0001 |  |  |  |  |
| *Brevibacterium epidermidis* |  | 0.02 |  |  |  |  |
| *Siphonobacter* |  |  | 2.20 *10 ^-7^ |  |  |  |
| *Pseudomonas otitidis* |  | 0.015 |  |  |  |  |
| *×[Eubacterium] nodatum group* |  |  |  | 0.009 |  |  |
| *Ezakiella* |  | 0.0001 |  |  |  |  |
| *Bradyrhizobium* |  | 1.40 *10^-8^ |  |  |  |  |
| *Burkholderia-Paraburkholderia* |  |  |  |  | 0.017 |  |
| *Burkholderia-Paraburkholderia* |  |  | 0.002 |  | 0.009 |  |
| +*Salmonella* |  |  |  |  |  | 0.009 |
| *Perlucidibaca* |  |  | 2.58 *10^-14^ |  |  |  |
| *Anaerococcus* |  | 0.013 |  |  |  |  |
| *Ezakiella* |  | 0.016 |  |  |  |  |
| ^+^*Citrobacter* |  |  |  |  |  | 8.07 * 10^-10^ |
| *Peptoniphilus* |  | 0.016 |  |  |  |  |
| *Pseudomonas* |  | 6.62 * 10^-26^ |  |  |  |  |
| *Pantoea* |  |  |  |  | 0.042 |  |
| *Sphingomonas* |  | 0.0002 |  |  |  |  |
| ^×+^*Sphingomonas* |  | 0.0001 | 0.001 | 0.021 | 0.018 | 0.015 |
| *Lawsonella* |  | 0.031 |  |  | 0.03 |  |
| *Comamonadaceae* |  | 0.002 |  |  |  |  |
| *Gluconobacter* |  |  |  |  | 0.042 |  |
| *Rhodococcus* |  | 0.005 |  |  |  |  |
| *Burkholderia-Paraburkholderia* |  | 0.04 | 0.039 |  |  |  |
| *Hafnia-Obesumbacterium* |  | 0.016 |  |  |  |  |
| *Tsukamurella* |  | 0.046 |  |  |  |  |
| *Enterobacter* |  |  | 0.009 |  |  |  |
| *Bacillus* |  |  |  |  | 0.016 |  |
| *Rothia* |  |  | 0.045 |  |  |  |
| OTUs highlighted in gray significantly co-varied between mosquito midguts and larval water  OTUs highlighted in yellow significantly co-varied between mosquito midguts and sugar pads  OTUs highlighted in cyan significantly co-varied between both midguts and larval water and between midguts and sugar pads  × OTUs that significantly co-varied between both midguts and larval water, but differed by mosquito species  ^+^ OTUs that significantly co-varied between both midguts and sugars, but differed by mosquito species | | | | | | |

**Figure Legends**

**Supplementary Figure S1**. Venn diagrams showing the overlap between OTUs in (A) the larval waters of independently reared *An* (setup 1, as per Figure 1) and *Ae* (setup 1) larvae as well as those of the co-reared species (setup 2), (B) the sugars of independently reared *An* (setup 1) and *Ae* (setup 1) adults as well as those shared by the two species (setup 3) ,and (C) the guts of *An* and *Ae* in the 3 different setups taken together.


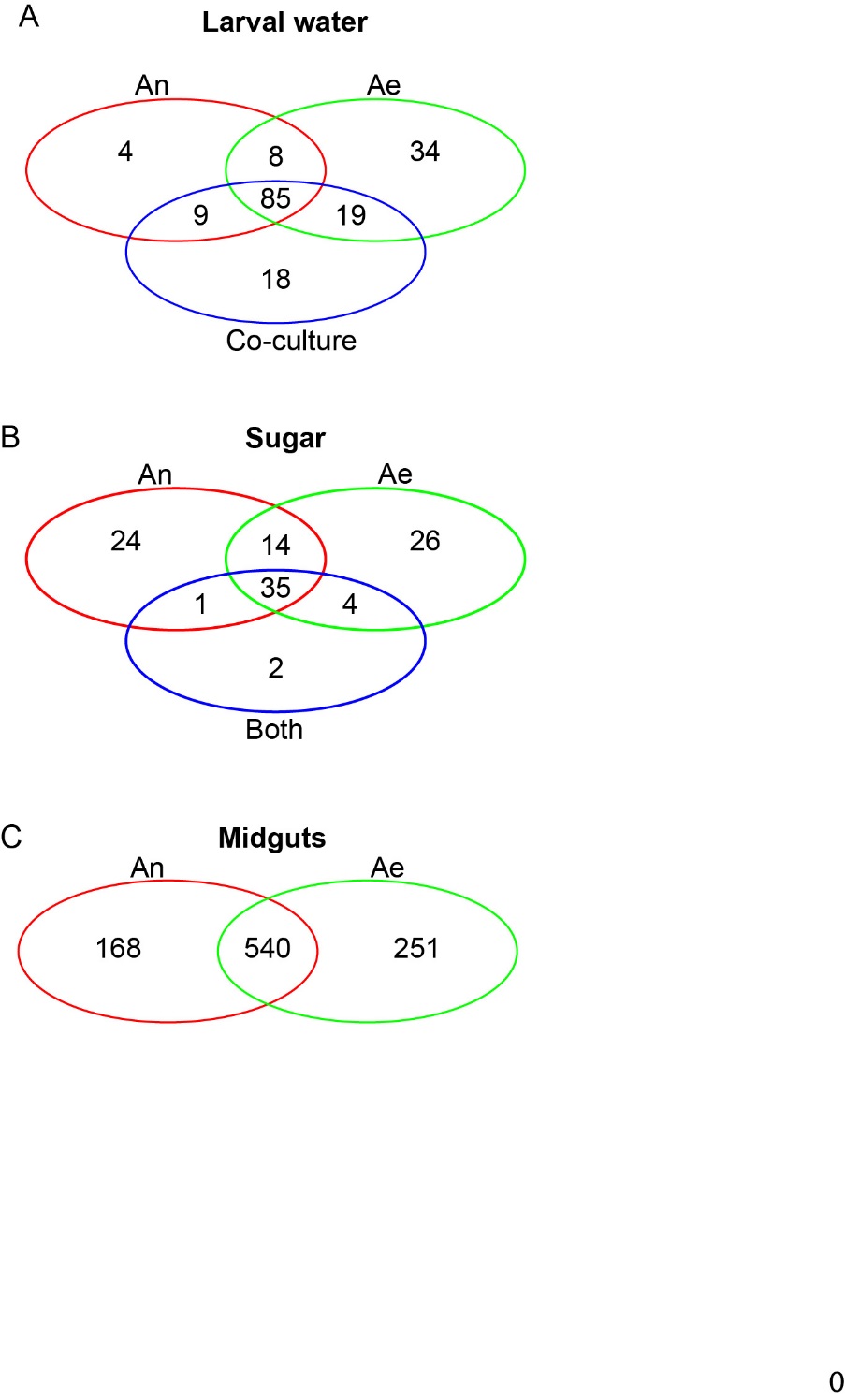

Supplement: Supplementary file 1 — Supplementary information. [file 41598_2020_60075_MOESM1_ESM.docx]
